# Mesoporous Bioactive Glass: Preparation, Characterisation, and Emerging Applications in Regenerative Medicine and Dentistry

**DOI:** 10.1016/j.identj.2026.109454

**Published:** 2026-02-25

**Authors:** Bakhtawar Mobeen, Nawshad Muhammad, Minati Choudhury, Ayesha Feroz, Sandleen Feroz

**Affiliations:** a Department of Dental Materials, Institute of Basic Medical Sciences, Khyber Medical University, Peshawar, KPK, Pakistan; b Division of Restorative Dentistry, School of Dentistry, IMU University, Bukit Jalil, Kuala Lumpur, Malaysia; c Department of Applied Bioinformatics, Eberhard Karls University of Tubingen, Germany; d School of Dentistry, The University of Queensland, Herston, QLD, Australia

**Keywords:** Mesoporous bioactive glass, Biomaterials, Enamel remineralisation, Regeneration, Drug delivery

## Abstract

**Introduction and Aims:**

Mesoporous bioactive glasses have emerged as advanced biomaterials due to their highly ordered mesoporous structure, large surface area, and enhanced biological reactivity. These properties distinguish them from conventional bioactive glasses and underpin their growing interest in regenerative medicine and dentistry. This review aims to summarise the development, synthesis, structural characteristics, and applications of mesoporous bioactive glasses, with a focus on their translational potential.

**Methods:**

A narrative review of the literature was conducted using major scientific databases to identify peer-reviewed studies related to the preparation, characterisation, and biomedical and dental applications of mesoporous bioactive glasses. Relevant in vitro and in vivo studies were critically analysed to synthesise current evidence and emerging trends.

**Results:**

Mesoporous bioactive glasses exhibit tunable pore sizes, high surface area, and enhanced ion and drug release capabilities, resulting in rapid hydroxyapatite formation and improved cell adhesion and proliferation. Various synthesis approaches, including sol–gel and templating techniques, allow precise control over composition and mesostructure. Reported applications include bone and soft tissue regeneration, drug delivery, wound healing, and antimicrobial therapies. In dentistry, mesoporous bioactive glasses have been explored for enamel remineralisation, periodontal regeneration, dentin hypersensitivity management, orthodontic applications, endodontic therapy, and implant surface modification. Most evidence remains pre-clinical.

**Conclusion:**

Mesoporous bioactive glasses have emerged as a promising class of biomaterials for regenerative and dental applications. However, limitations related to mechanical strength, manufacturing scalability, and the lack of clinical studies currently restrict widespread clinical applications.

**Clinical Relevance:**

Mesoporous bioactive glasses offer enhanced bioactivity and therapeutic delivery compared with conventional bioactive glasses, highlighting their potential to improve regenerative and restorative dental outcomes once translational challenges are addressed

## Introduction

Regenerative biomaterials have become central to modern regenerative medicine due to their ability to interact with biological tissues and actively promote repair and regeneration.^1^ Among these, bioactive glasses (BAGs) represent a unique class of synthetic biomaterials capable of forming a direct chemical bond with living tissue.^2^ BAG is a non-crystalline ceramic, which mimics the porous nature of the bone with regeneration and revascularisation ability.^2^^,^^3^ The development of bioactive glass dates back to 1969, when Hench and colleagues introduced the first bioactive composition, 45S5 Bioglass, composed of 45 wt% SiO₂, 24.5 wt% Na₂O, 24.5 wt% CaO, and 6 wt% P₂O₅.^4^ This material marked a paradigm shift from passive, bio-inert implants to bioactive materials that chemically interact with surrounding tissues to promote regeneration transition.^2^^,^^3^ Furthermore, Bioglass which was majorly silica based became the reference formulation for subsequent silicate-, borate- and phosphate-based bioactive glasses and their clinical products.^5^ From a compositional perspective, they are therefore commonly classified into silicate-based, borate-based, and phosphate-based bioactive glasses, each with distinct degradation behavior, ion-release profiles, and applications in bone regeneration.^6^ All these glasses share the ability to form a bone-like interfacial layer and exhibit osteoconductive and, in many cases, osteoinductive properties, enabling strong bonding with both hard and soft tissues and supporting angiogenesis and wound healing. Over the following decades, sol–gel routes were developed to increase specific surface area and silanol density, and compositional modifications were investigated to modify ion release, degradation behaviour and therapeutic functions, but these materials still lacked ordered mesoporous structure.^7^

A major advancement was achieved by Vallet-Regí and colleagues in the early 2000s, when surfactant-templated mesoporous silica chemistry was translated into biomedicine, first through drug-releasing ordered mesoporous silicas.^5^ Based on this work, several other studies have been reported that introduced CaO- and P-containing mesoporous bioactive glasses (MBGs) in the SiO_2_–CaO–P_2_O_5_ system, synthesised by sol–gel methods coupled with evaporation-induced self-assembly using non-ionic surfactants (for example, Pluronic P123) as structure-modulating agents, yielding highly ordered mesoporous frameworks with very high surface areas and narrow pore size distributions that enhance apatite formation and provide versatile platforms for loading therapeutic ions, drugs and biomolecules.^6^, ^7^, ^8^ In this evolutionary framework, the original 45S5 Bioglass introduced in 1969 represents the foundational discovery of bioactivity in melt-derived silicate glasses, whereas MBGs represent a transformative third generation of bioactive materials. Through their precisely engineered meso-porous structures, they transcend the passive role of traditional bioactive glasses, offering a versatile and tunable platform for advanced regenerative and therapeutic applications.

MBGs is a third generation bioactive glass, with a highly organised pore structure containing mesopores, ranging in diameter from 2 to 50 nanometers.^9^ These structural features are achieved through surfactant-directed sol–gel synthesis, which enables the formation of long-range ordered structures such as two-dimensional hexagonal or three-dimensional cubic arrangements. The resulting architecture significantly enhances ion exchange kinetics and accelerates hydroxyapatite formation compared with non-mesoporous bioactive glasses, owing to the increased density of surface silanol groups.^10^^,^^11^ In addition, the well-defined mesoporous network enables high loading capacities and controlled release of therapeutic ions and bioactive molecules. Overall, these attributes confer superior bioactivity, osteoconductivity, and multifunctionality to MBGs, positioning them as advanced and versatile platforms for bone regeneration and localised therapeutic delivery as shown in Table 1. This review aims to discuss the structure, synthesis, properties, and applications of mesoporous bioactive glass.

**Table 1:** Studies reporting role of MBG in bone tissue engineering.

Author’s name	Year of publication	Type of study	Mesoporous bioactive glass	Preparation technique	Role of MBG
Sesha et al.^54^	2023	In vitro and in vivo	Scaffold	Sol gel method	Simvastatin-containing MBG and disulfide molybdenum composite scaffolds offer a possible alternative to the limitations of conventional bone grafting procedures
Maria et al.^55^	2023	In vitro	Glass powder composite	Modified stober process	Pagnum harmala loaded MBGNs have the potential to serve as scaffold or a component in the coatings for bone tissue engineering applications
Jiang feng et al.^48^	2022	In vivo	Scaffold	Solvent casting leaching technique	MBG/ PGA-PCL scaffolds can be used as potential biomaterial for bone repair with highly, bioactivity, biodegradability and osteogenic activity
Jing chen et al.^49^	2023	In vivo and in vitro	Scaffold	Self-assembly method induced by volatilisation	Tetramethylpyrazine and icariin loaded MBG scaffold may encourage osteogenesis and angiogenesis in bone regeneration
Irina Atkinson et al.^51^	2022	In vitro	Scaffold	Templating	Scaffolds derived from cerium-containing mesoporous bioactive glasses show potential for bone regeneration
Lorena García et al.^50^	2024	In vivo	Scaffold	Evaporation induced self-assembly	MBG scaffold by itself and enhanced with osteostatin promote formation of bone compared to the unfilled defect
A. Maha et al.^56^	2025	In vitro	Powder	Sol gel microemulsion technique	Tb ion doped MBGs are ideal for bone tissue regeneration. A promising material for enhanced bone repair and regenerative procedures
Zoleikha et al.^57^	2023	In vitro	Viscogel	Sol gel process	Copper and cobalt doped borate MBG's showed significant potential in bone tissue engineering applications
M.Moll et al. ^58^	2024	In vitro	Powder	Sol gel process	Mesoporous bioactive glass nanoparticles containing molybdenum have pro-osteogenic and pro-angiogenic properties
Haoli et al.^59^	2025	In vitro and in vivo	Hydrogel	Stober method	PCL scaffolds loaded with magnesium/alginate-doped mesoporous bioactive glass hydrogel promotes in situ tissue regenration in intricate intrajoint degenerative microenvironments
Ruibang et al.^60^	2023	In vitro and in vivo	Hydrogel	Templating	MBG/sodium alginate hydrogel laden with melatonin can successfully reduce inflammation, which is anticipated to encourage intervertebral disc regeneration
Ricardo J et al.^46^	2023	In vitro	Composite	Sol gel technique	Polyvinylpyrrolidone loaded mesoporous bioactive glass has ability to induce osteogenesis and angiogenesis
Akrity et al.^47^	2023	In vitro	Powder	Sol gel method	Addition of Cu and Mg ions in MBG promote bone regeneration because of their bioactivity, proliferation of cells, and antibacterial properties
Ying Zhang et al.^61^	2023	In vitro	Microspheres composite	Sol gel method	Erbium and ytterbium containing MBG has potential as an upconversion biomonitoring material for bone tissue engineering
Parichart et al.^62^	2024	In vitro	Powder	Sol gel technique	Zinc and strontium Co substituted MBG Nanoparticles promotes the bone regeneration
Ying Zhang et al.^63^	2021	In vitro	Powder nanoparticles	Sol gel method	Se and Te doped MBGs have potential for treating malignant bone tumors through local drug delivery and anticancer capabilities in bone tissue engineering

## Methodology

A comprehensive literature search was conducted using electronic databases including PubMed, Scopus, and Medline. Articles published between 2018 and 2025 were considered to ensure inclusion of recent and relevant advances in the field.

The search strategy employed combinations of the following keywords: *mesoporous bioactive glass, biomaterials, enamel remineralisation, regenerative medicine, drug delivery*, and *bone regeneration*. Additional relevant articles were identified through manual screening of reference lists from selected publications.

Studies were included based on their relevance to the synthesis, characterisation, functional properties, and biomedical applications of mesoporous bioactive glasses. Both original research articles and review papers published in peer-reviewed journals were considered. Studies focusing on in vitro and in vivo investigations were included to provide comprehensive insight into biological performance. Exclusion criteria comprised non-English publications, conference abstracts without full manuscripts, articles lacking sufficient experimental details, and studies not directly related to mesoporous bioactive glass systems. The selected literature was critically analysed to provide an integrated overview of current advances, emerging trends, and future directions in the field.

## Structure, properties, and synthesis of MBG’S

### Structure

MBGs are distinguished by mesopores that range in size from 2 to 50 nm and particle sizes between 20 and 800 nm^9^. On the basis of pore ordering and arrangement mesoporous bioactive glass can be hexagonal (2D), cubic (3D), and disordered. Mesoporous bioactive glasses are mostly silicate-based, but they can also contain other oxides, such as phosphate-based, boron-based, and doped MBGs.^12^

The mesoporous channels enhance their textural qualities, reactivity, and bioactivity, and their enormous pore volume permits the simultaneous loading of growth factors, medications, and other bioactive compounds.^11^ High loading and controlled release of therapeutic ions are made possible by the ultrahigh surface area (>100 m^2^/g) and highly organised mesostructured channels. Additionally, their nanometric size makes it easier for cells to internalise them, creating new avenues for targeting intracellular bacteria.^12^^,^^13^

### Properties

Because of their high pore volume, high drug loading capacity and flow characteristics, they have been utilised as bioactive fillers in composite biomaterials or injectable biomaterials.^14^ It provides certain biological capabilities (such as angiogenesis, osteogenesis, anti-inflammatory response, and antibacterial action), because of therapeutic ions added to the silica network.^15^

**Fig. 1:**
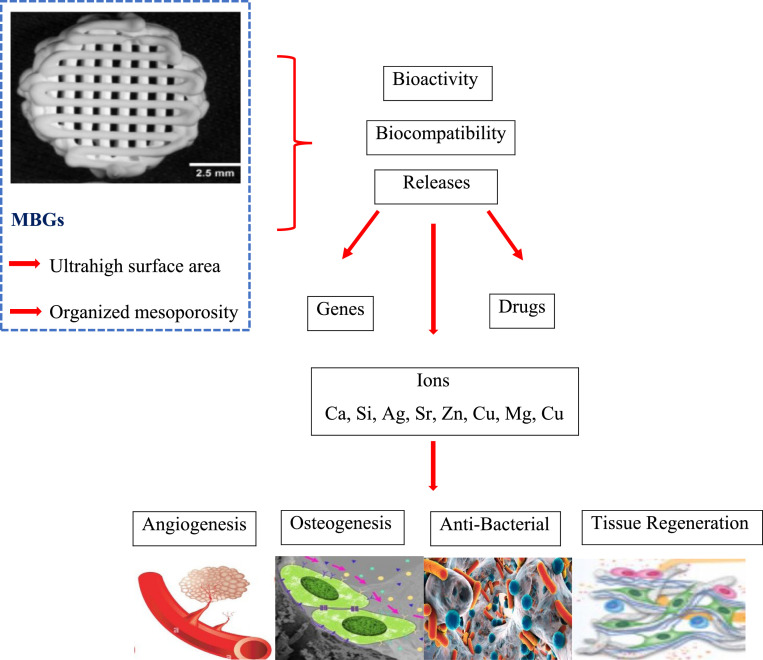
Shows various properties of mesoporous bioactive glas.

Recently, mesoporous bioactive glass that are doped with therapeutic ions have emerged as novel multifunctional systems capable of delivering ions and drugs simultaneously, hence producing synergistic effects.^16^ They can interface with biomolecules and polymeric matrices to a greater degree than nonporous bioactive glass, potentially leading to increased mechanical strengthening and biological capabilities.^17^ They can be loaded with different drugs or metallic ions to facilitate bone tissue engineering, targeted drug delivery, and wound healing.^18^ When compared to dense bioactive glass particles of the same composition, MBGNs' superior textural qualities greatly increase HCA production in interaction with biological fluids, hence promoting apatite mineralisation. Additionally, their pore capacity and specific surface area offer benefits for loading and delivering various medications (such as antibiotics and anti-cancer treatments)^18^ and therapeutically bioactive molecules such as genes and growth factors that enhance or modify their intrinsic pro-osteogenic properties.^19^^,^^20^ Mesoporous bioactive glass materials, which are based on the ternary system SiO 2-CaO-P 2 O-5, have been extensively studied for their ability to treat bacteria and replace missing bone.^21^ Properties of mesoporous bioactive glass are shown in Figure 1.

### Synthesis

MBGs are produced commonly either by the sol gel method or by using template technique.

#### Sol-gel method

A common technique for creating mesoporous bioactive glasses (MBGs) is the sol-gel process. In this procedure, a colloidal solution (sol) is prepared, then it gels to create a solid network. The technique works especially well for creating materials with high porosity, superior bioactivity, and molecular-level composition control.^22^

Sol is primarily produced from precursor compounds, which are usually salts or metal alkoxides.^12^ For the silica component common precursors include tetra-methyl orthosilicate or tetraethyl orthosilicate. Calcium nitrate for calcium component. Phosphorus pentoxide for the phosphorus component.^23^ These precursors are dissolved in an alcohol solvent to produce a uniform solution. A catalyst (usually an acid like hydrochloric acid or a base like ammonia) is added to facilitate hydrolysis and condensation reactions. The sol proceeds through hydrolysis, in which the metal alkoxides react with water to generate alcohols and metal hydroxides.^24^ The hydroxyl groups subsequently condense and create bonds with metal ions, generating a gel-like structure. The sol slowly turns into a gel as the condensation process advances. During gelation, the sol creates a 3-dimensional network that traps solvent and precursor molecules. After gelation, the wet gel is carefully dried to eliminate the solvent, in an oven at a mild temperature (usually 50-100°C).^24^ After drying, the gel is calcined at a higher temperature. This stage eliminates any remaining organic residues, encourages more condensation, and crystallises the network, resulting in a solid bioactive glass.^24^

The sol-gel process allows for the production of very porous materials with increased surface area, complete control over the stoichiometry of mesoporous bioactive glass, allowing for fine tuning of composition and features while maintaining the material's bioactivity as ashown in Table 2.^10^ The molecular scale blending of precursors promotes equal distribution of bioactive components, which improves the overall performance of MBGs. Sol-gel processing approach creates structures with greater osteogenic potential and higher bioactivity because of greater surface area, intrinsic porosity, and more surface hydroxyl groups compared to the traditional melt-derived process.^22^

**Table 2:** A comparison of different aspects of the sol-gel process with templating technique.

Features	Sol-Gel method	Templating technique
Final product	Homogenous glass	Ordered mesoporous structure
Surface area	Moderate to high	High
Order of mesopores	Arbitrary	Ordered
Pore regulation	Limited	Highly controlled
Complexity	Simple	More complicated
Bioactivity	High	Very high
Cost	Low	High
Application	Coatings, bone fillers	Drug delivery, scaffolds and tissue engineering
Limitation	Shrinkage and cracking can occur during drying	Requires removal of template

### Limitations of sol gel method

• Improper control of pore size and arrangement.
• Prolong drying time and aging.
• Cracking and shrinkage during calcination and drying.
• Challenges in scaling up the process for large-scale or industrial production

#### Preparation via templating technique

One popular method for creating mesoporous bioactive glasses is the templating technique. A flexible technique for managing the shape and composition of bioglass is the templated sol-gel scaffold as shown in Table 2. In order to create a porous scaffold, this procedure entails foaming the sol with the help of a surfactant and then undergoing condensation and gelation reactions.^25^ The scaffold's pore architecture is largely controlled by the usage of templates. The scaffold's surface characteristics are also significant since they have the potential to influence how molecules and ions are distributed.^26^ Using an organic or inorganic template, this technique directs the development of mesoporous structures with specific pore size, shape, and distribution. A network of pores remains after the mesoporous structure is produced and the template is removed. There are two types of templating approaches: soft templating and hard templating.

#### Hard templating (inorganic template)

In hard templating, the mesoporous structure is molded using a solid template, typically composed of inorganic elements. Usually, etching or a chemical dissolvate is used to remove the template once the mesoporous material has formed around it with exact control over pore size, hard templates can produce highly organized mesoporous structures. The drawbacks of using a hard template are, it can be difficult to remove the template, especially from deep pores and could not be completely removed.^25^

#### Soft templating (organic template)

Soft templating is a process in which templates self-assemble in solution to create micelles or other nanostructures that direct the final material's mesopore development. Soft templates are frequently made of block copolymers (like Pluronic) or surfactants (like cetyltrimethylammonium bromide).^22^ The organic surfactant template is eliminated by calcination at high temperatures (usually 500–700°C) or by washing with solvents following the gelation and drying procedures. Mesoporous structures are left behind by this procedure. Compared to hard templating, soft templating is typically carried out in milder environments with a lower chance of causing damage to the finished product. Although mesoporous structures can be formed using soft templating, they may not necessarily be as highly ordered as those produced by hard templating. This is one of its drawbacks.

### Limitations of templating technique

• More complex synthesis procedures compared with conventional sol–gel processing.
• Requirement for template removal, typically through solvent extraction or high-temperature calcination.
• Higher overall cost due to the use of structure-directing agents and surfactants as shown in Table 2.
• Greater challenges in process scalability compared to traditional sol–gel methods.

### Doping of Mesoporous bioactive glass particles

Doping refers to the incorporation of additional elements into the bioactive glass network to modify its chemical, biological, and physical properties.^18^ In mesoporous bioactive glasses (MBGs), ion doping is primarily employed to enhance bioactivity, biocompatibility, mechanical performance, and to introduce specific therapeutic functions.^27^ A wide range of biologically relevant ions including strontium (Sr²⁺), calcium (Ca²⁺), magnesium (Mg²⁺), zinc (Zn²⁺), copper (Cu²⁺), silver (Ag⁺), boron (B), phosphorus (P), and molybdenum (Mo) has been successfully incorporated into MBGs.^15^^,^^27^

**Fig. 2:**
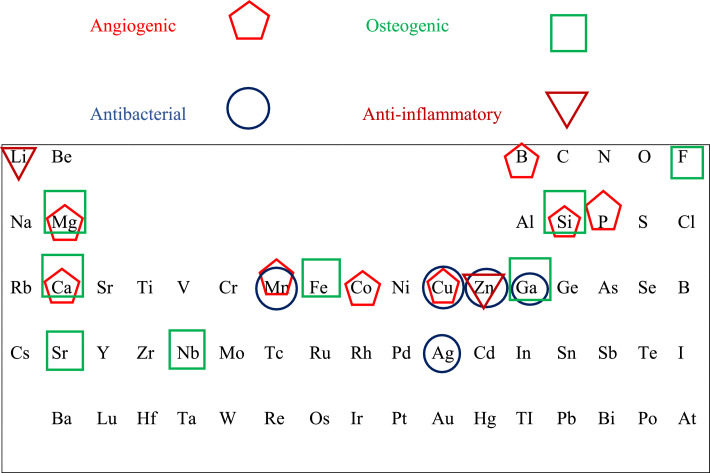
Elements included in the mesoporous bioactive glass and their biological activity.

These dopants influence cell–material interactions, promote tissue adhesion, and support healing and regeneration through controlled ion release.^10^ For instance, copper- and silver-doped MBGs impart antibacterial activity, while strontium, zinc, and molybdenum are associated with enhanced osteogenic differentiation and matrix mineralisation.^15^ The incorporation of copper-doped mesoporous bioactive glass nanospheres into dental resin composites has also been shown to improve remineralisation potential when combined with inert silica and glass microfillers. Overall, metallic-ion doping enables the development of multifunctional MBGs capable of simultaneously promoting osteogenesis and antimicrobial activity.^13^^,^^28^ A summary of non-toxic dopant elements and their associated biological functions is presented in Figure 2.

#### Functionalisation of Mesoporous bioactive glass

The process of chemically altering MBGs' surface or structure to add particular bioactive groups and enhance their interactions with biological surroundings is known as functionalisation.

MBGs can have functional groups grafted onto their surface, including amine, carboxyl, phosphonic acid, and silanol groups.^29^ These groups can enhance cell adhesion, encourage osteoblast development, or even facilitate the attachment of bioactive substances such as medications or growth factors.^28^ MBGs' mechanical qualities can be improved and a more regulated release of ions or medications can be achieved by coating them with biodegradable polymers (such as PCL or PLGA).^30^ Nanoparticles (such as graphene or carbon nanotubes) can provide the glass new functionality while also enhancing its mechanical qualities and bioactivity. The abundance of free silanol groups on MBG surfaces creates an intriguing pathway for organisms with antibacterial qualities to anchor via covalent attachment.^21^ Functionalised MBGs can enhance osteoconductivity, stimulate bone formation, act as a medication delivery mechanism, and stimulate bone cell proliferation (osteoblasts). Functionalisation with antimicrobial agents (eg, silver nanoparticles or antibiotics) can give MBGs antibacterial characteristics, lowering infection risk in tissue scaffolds or bone implants.^21^

Doping modifies the material's intrinsic qualities, whereas functionalisation modifies surface and interfaces to improve biological interactions. In combination, these technologies allow the construction of next-generation mesoporous bioactive materials that can enhance healing process, stimulate tissue regeneration, and provide controlled releases of drugs, making them appropriate for use in the repair of bones, tissue engineering, and various other biomedical sectors.^27^

## Applications of MBG and its composite in regenerative medicine

**Fig. 3:**
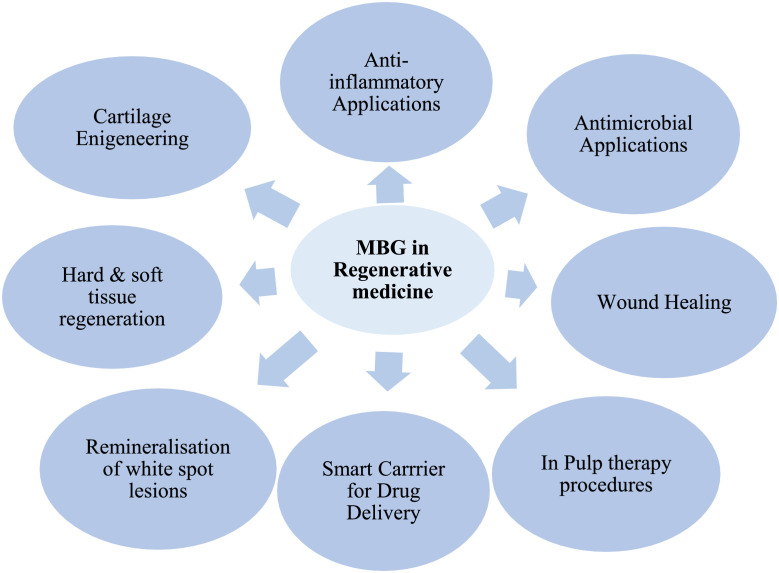
Applications of mesoporous bioactive glass in various fields of biomedicine.

Recently there has been a dire interest in the application of MBG in regenerative medicine due to its good bioactivity, biocompatibility, tunable pore structure, and capacity to activate biological responses.^7^^,^^27^ MBGs-based hierarchical scaffolds and systems have been discovered and utilised in tissue engineering, drug delivery, tumor therapy, biosensing, and other fields. Various growth factors, including BMP, VEGF, TGF-β and brain-derived neurotrophic factor (BDNF), are loaded to promote bone regeneration and fracture healing. MBGs have potential as gene delivery methods for medical treatment.^31^ It has potential as gene delivery platforms for disease treatment, genome function and transfer of biological functional nucleic acids into target cells, their expression inside cells, and the achievement of particular functions. MBGs and composite materials have showed promise in speeding hemostasis and limiting infection due to their specific properties, which include porous architectures, blood clot-promoting surfaces, and quick plasma absorption.^27^ MBGs show significant promise for cancer therapy because to their unique qualities, of targeting and regulating anti-tumor medication delivery, and being doped with anti-tumor and magnetic ions.^28^ Figure 3 provides an overview of the key application domains of mesoporous bioactive glasses in regenerative medicine, including tissue engineering, drug delivery, and therapeutic platforms.

### Applications in dentistry

Mesoporous bioactive glasses (MBGs) have been investigated for a broad range of dental applications, including restorative dentistry, orthodontics, endodontics, implantology, and the management of dentin hypersensitivity.^32^

At present, most commercially available dental products employ conventional melt-derived bioactive glasses rather than mesoporous formulations; however, MBG-based systems are increasingly being explored at the pre-clinical and early translational stages due to their enhanced surface area, ion-release capability, and drug-loading potential.^33^

#### Restorative dentistry and dentin remineralisation

MBGs have shown considerable promise as functional additives in restorative materials owing to their ability to promote remineralisation and modulate local pH.^32^^,^^34^ Their incorporation into dental adhesives and resin composites enhances apatite formation, occludes dentinal tubules, and supports the repair of demineralised enamel and dentin.^35^ MBG-containing universal adhesives have demonstrated efficacy comparable to, or exceeding, conventional desensitizing agents, suggesting their potential use as in-office treatments for dentin hypersensitivity.^36^ In addition, MBGs loaded with amorphous calcium phosphate have shown improved remineralisation of enamel white-spot lesions, highlighting their clinical relevance in preventive and restorative dentistry.^37^

#### Orthodontic applications

In orthodontics, MBGs have been incorporated into self-adhesive resins and bonding agents to reduce enamel demineralisation around brackets while maintaining adequate bond strength.^38^ MBG-based orthodontic adhesives exhibit buffering capacity in acidic oral environments, thereby limiting enamel mineral loss in areas prone to white-spot lesion formation. The inclusion of mesoporous bioactive glass nanoparticles has also been shown to enhance antibacterial activity and mechanical performance, contributing to improved clinical outcomes during orthodontic treatment.^38^^,^^39^

#### Endodontics and pulp therapy

MBGs have been explored as intracanal medicaments and pulp-capping materials due to their antibacterial properties and bioactivity.^40^ Sol–gel-derived bioactive glasses containing bactericidal ions have demonstrated effective inhibition of *Enterococcus faecalis* and *Candida albicans*, pathogens commonly associated with endodontic treatment failure.^41^^,^^42^ Furthermore, calcium silicate cements incorporating mesoporous bioactive glass nanoparticles have shown improved cell proliferation, mineralised matrix formation, and antibacterial performance, supporting their potential use in vital pulp therapy.^43^

#### Dental implants and peri-implantitis

Surface modification of dental implants using MBG-based coatings has been investigated to enhance osteointegration while reducing bacterial colonisation.^44^ Silver-containing mesoporous bioactive glass films exhibit good biocompatibility and sustained antimicrobial activity, offering a promising strategy for preventing early implant failure and peri-implantitis.^45^ The controlled release of bioactive and antimicrobial ions from MBG coatings supports both bone formation and infection control at the implant–tissue interface. A surface modification method for titanium implants using HMDSZ, oxygen plasma, and grafting of a thermosensitive hydrogel demonstrated antibacterial performance and enhanced osteointegration.^44^ The incorporation of hydroxyapatite nano particles in dental implant procedures promotes the remineralisation and accelerates the process of osseointegration.^37^

#### Commercial translation and current limitations

Despite encouraging pre-clinical outcomes, the clinical translation of MBGs in dentistry remains limited. Currently marketed desensitizing and remineralizing products, such as NovaMin and BioMin F, are based on conventional melt-derived bioactive glasses rather than mesoporous formulations.^33^ While MBGs offer superior textural and functional properties, challenges related to manufacturing scalability, regulatory approval, and mechanical reinforcement must be addressed before widespread clinical adoption can be achieved.^30^

### Applications in bone tissue engineering

Electrospun polyvinylpyrrolidone (PVP) nanofibers with mesoporous bioactive glass demonstrated the composites potential for use in bone tissue engineering. The Saos-2 cell line showed no signs of cytotoxicity from the composite. Because they combine the high bioactivity of MBG 80S15 nanosised powder and the advantage of electrospun PVP mats' flexibility, electrospun nanocomposite fiber mats show promise as bone scaffolds.^46^ Addition of Mg and Cu ions in MBG at lower concentrations of up to 2 mol% can be beneficial in bone regeneration due to their bioactivity, proliferation of cells, and antibacterial properties. Mg and Cu (up to 2 mol%) in the MBG system may be essential for promoting the development of new tissue through the delivery of Cu and Mg ions throughout the bone regeneration process. The new Mg and Cu co-doped MBGs show promise as multipurpose fillers that are bioactive and antimicrobial.^47^

The PGC/M10-40 poly(caprolactone-co-glycolide) scaffolds demonstrated superior osteogenic activity, cytocompatibility, degradation, hydrophilicity, and bioactivity. By incorporation of MBG, these PGC/M scaffold features might be modified to accommodate various bone-repairing situations. These findings demonstrated that the MBG/PGA-PCL composite is a highly adjustable platform for the creation of bone substitutes, offering customised osteogenic and biodegradation properties to address a range of bony repair scenarios.^48^ Scaffolds containing MBG that are additively created will surely gain value in order to promote the safe and effective healing of bone deformities.^49^ MBG scaffold by itself, or enhanced with bone marrow aspirate or osteostatin, increases bone growth as compared to unfilled defects. The study demonstrated that it is possible to cure important bone abnormalities by combining MBGs with osteogenic peptides such as osteostatin, with promising prospects for conversion into clinical practice.^50^

Cerium-containing MBG-derived biomaterial scaffolds demonstrated significant cytocompatibility in the fibroblast cell line of mouse (NCTC clone L929). These scaffolds are promising prospects for bone tissue engineering applications based on their antibacterial potential, bioactivity, and controlled drug delivery activities.^51^ The injectable mesoporous bioactive glass/fibrin glue composite hydrogel greatly increased formation of bone within the expanded mid-palatal suture, reduced osteoclastogenesis, and improved the proportion of remodeling of bone favouring osteogenesis. After rapid maxillary extension, a combination of injectable biomaterial and retainers was shown to be a potential treatment to improve osteogenesis. This composite hydrogel production may offer fresh perspectives on treating craniomaxillofacial deformities and designing bone-repairing biomaterials with increased regenerative efficiency.^52^ Gentamicin-impregnated mesoporous bioactive glass calcium phosphate bone cement exhibits good bacteriostatic action and does not considerably delay CPC response, making it a great option for bone substitute substances.^53^ The table below outlines the numerous applications of mesoporous bioactive glass in bone tissue engineering, with a focus on drug delivery, enhancing osteogenesis and scaffold advancement.

## Limitations

Number of papers on mesoporous bioactive glass has increased dramatically in recent years, there are still issues that need to be resolved before these bio materials may be used in clinical trials.^64^ The intrinsic brittleness of MBGs limits their use in load-bearing applications, mandating their conjunction with polymers or other supporting materials. Concerns have also been raised about MBGs' breakdown, long-term stability, and ion release kinetics in physiological settings, which are yet not fully known.^65^ Furthermore, the majority of investigations are limited to in vitro testing or short-term in vivo experiments, with few thorough long-term animal studies and clinical trials. Variability in pore size, composition, and surface functionalisation across research studies make comparisons between them difficulty.^30^

Mesoporous bioactive glasses (MBGs) have significant manufacturing and mechanical performance limitations as compared to conventional melt-derived bioactive glasses.

Its dependence on multiple steps sol-gel and surfactant-templating processes challenges large-scale manufacturing and lowers reproducibility when compared to traditional melt-quenching approaches. Furthermore, MBGs' highly porous geometry leads to lower mechanical strength and fracture resistance even though it is advantageous for improved bioactivity and delivery of drug.^65^ The use of MBGs in load-bearing situations is currently restricted by these issues, which require composite or reinforcing techniques for translation into clinical practice.

## Conclusion and future directions

Biomedical applications of mesoporous bioactive glass have achieved significant growth in recent decades because of its ease of use and versatility in controlling its morphology and properties. It represents a better advancement in the field of regenerative medicines and biomaterials. The addition of therapeutic ions, drugs and growth factors into the MBG structure opens up a number of possibilities for designing adaptable and customised bio ceramics that meet the clinical standards of bone tissue engineering, soft tissue repair and infection control. Recent research indicates that MBGs possess significant potential in stimuli-responsive drug delivery, particularly through the precise regulation of drug release by chemical changes and nanostructural innovations.

Future research needs to focus on designing multifunctional and stimulus-sensitive MBGs and combining them with advanced fabrication techniques like 3D printing and nanocomposites to construct next-generation regeneration technologies.

## Funding

This research was supported by a research grant from the Australian Society of Endodontology Inc.

## CRediT authorship contribution statement

**Bakhtawar Mobeen:** Writing – original draft, Writing – review & editing. **Nawshad Muhammad:** Supervision, Writing – review & editing. **Minati Choudhury:** Writing – review & editing. **Ayesha Feroz:** Writing – review & editing, Resources. **Sandleen Feroz:** Supervision, Writing – original draft, Writing – review & editing.

## Conflict of interest

None disclosed.
